# Conversion of the CD8 lineage to CD4 T cells

**DOI:** 10.18632/oncotarget.5235

**Published:** 2015-08-22

**Authors:** Maria P. Guzman, Zhibin Chen

**Affiliations:** Department of Microbiology and Immunology, University of Miami Miller School of Medicine, Miami, Florida, USA

**Keywords:** T lymphocyte, lineage, plasticity, immune tolerance, selfless

Mature T lymphocytes possess a CD4 or CD8 lineage-specific marker. These molecules also serve as a co-receptor which along with a specific T Cell Receptor (TCR) binds to MHC class II (MHCII) or class I (MHCI), respectively. MHCI is expressed by all nucleated cells, whereas MHCII by antigen-presenting cells (APC). CD8 T cells kill target cells through MHCI-based recognition. CD4 T cells recognize MHCII and act either as helper T (Th) cells potentiating immunity or as regulatory T (Treg) cells inducing tolerance.

**Figure 1:**
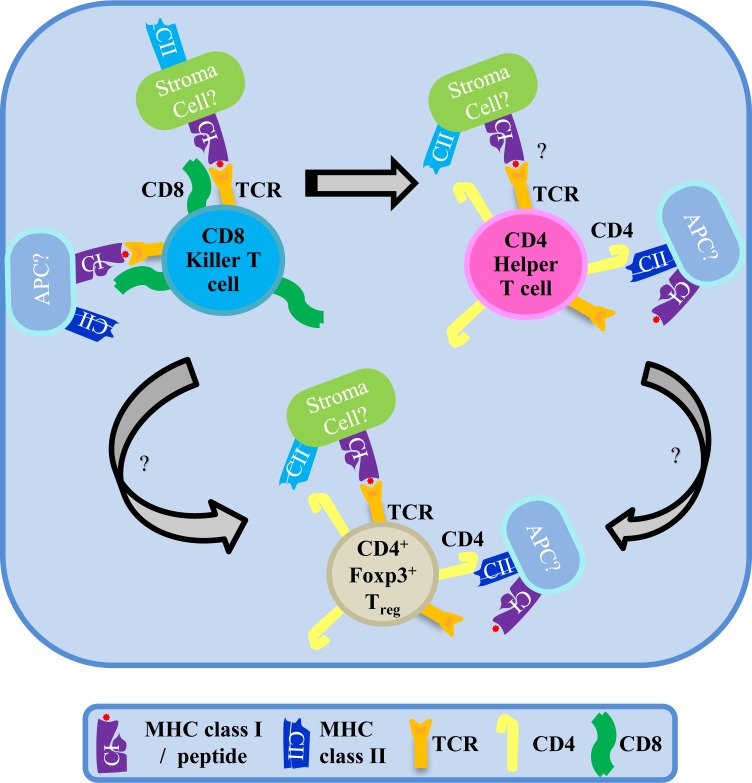
The mismatch to the rescue — a tale of the convert The CD8 killer T cell lineage converts to MHCI-restricted CD4 T helper cells and CD4^+^Foxp3^+^ suppressor cells in the gut-associated environment. Despite the mismatch of the CD4 co-receptor on the converted CD4 T cells to MHCI-restricted TCR binding, the host-intrinsic plasticity of the CD8 T cell lineage may serve as an alternative pathway to induce “selfless” tolerance and restore immune balance, especially at the interface with microbiota.

The development of the adaptive immune repertoire is based on discrimination of self-antigens in the host against nonself-antigens from foreign invaders [1]. This concept has been serving as a pillar for immunology but faces a challenge to accommodate the interplay between the host immune system and the mutualistic microbiota. We tracked the clonal fate of CD4 and CD8 T cells at the interface with gut microbiota. We found that CD8 T cells cross-differentiated into MHCI-restricted CD4 T_h_ cells or Foxp3^+^ T_reg_ cells [2] (Figure 1). The CD8 lineage plasticity was found in two independent models of TCR-trangenic mice (OT1 and 8.3) and natural CD8 T cells from wildtype mice. The conversion from CD8 T cells to MHCI-restricted CD4 T_reg_ cells occurred in the gut-associated environment without regard to self-antigens, with a host-intrinsic plasticity amplified by microbiota. The MHCI-T_reg_ cell, in its physiological niche in the gut lamina propria or in a setting of adoptive transfer, potently suppressed inflammatory damage even in the apparent absence of cognate antigens [2].

Why is such an intrinsic plasticity built in the host immune system during evolution? The T cell repertoire consists of CD4 and CD8 T cells generated by thymic selection in newborns. With age, the thymus ceases to function but the thymic-derived T cell repertoire largely persists. In certain diseases, a portion or a subtype of T cells might be lost. Conceivably, evolution may have endowed mechanisms to restore the immune balance to cope with catastrophic damages, such as T cell depletion by viral infections or natural irradiation. The plasticity of the CD8 T cell lineage elucidated by our study has perhaps evolved as an alternative pathway to protect the integrity of the adaptive immune system. In the adverse event of CD4 T cell depletion, CD4 populations can be replenished via cross-differentiation from CD8 T cells. One of the fascinating aspects of this transition is the requirement of MHCII despite that the clonotype TCR recognizes MHCI-presentated antigens [2].

How is the CD8-to-CD4 lineage plasticity relevant to biomedicine? In the modern world, HIV infection perhaps represents the most known example of catastrophic loss of CD4 T cells and subsequent imbalance of the CD8 versus CD4 lineages. It remains to be seen whether the depletion of CD4 T cells in HIV infection triggers cross-differentiation from the CD8 lineage to CD4 T cells. If so, the conversion might not only replenish the CD4 T cell pool with new targets for infection by HIV but also exacerbate the depletion of CD8 populations, while creating a potent immunosuppression in the gut mucosa by the converted MHCI-T_reg_ cells.

An imbalance of CD8 versus CD4 T cells could also be medically introduced in a type of cancer immunotherapy, wherein a patient is subject to immunoablation conditioning and then receives adoptive transfer of CD8 T cells enriched for specificities against tumors. Tumors present an immunoprivileged microenvironment with some neoantigens but mostly self-antigens [3]. It remains to be examined whether such a microenvironment triggers CD8-to-CD4 lineage conversion and generation of MHCI-T_reg_cells.

In autoimmune diseases, CD4 T cell depletion has been tested as a potential therapy. In our study, depletion of CD4 T cells in normal mice led to an increased population of CD4 T cells converted from the CD8 lineage. The converted CD4^+^Foxp3^−^ cells may help activation of CD8 T cells. Moreover, MHCI-T_reg_ cells might provide a potent antidote against autoimmune damage by CD8 T cells [2]. The conventional MHCII-restricted CD4^+^Foxp3^+^ T cells provide dominant tolerance through a variety of mechanisms [4], including cellular contact-based interaction between T_reg_ and pathogenic T cells [5]. One might envisage that MHCI-based recognition facilitates such a direct cellular contact. Of note, MHCI-restricted CD4 T cell clones exist in the natural repertoire of healthy humans [6]. A number of studies have also showed the possibilities of engineering human CD4 T cells recognizing MHCI-presented antigens by transducing MHCI-restricted TCR into CD4 T cells, including CD4 T_reg_cells [7]. Along this direction, understanding the cellular and molecular mechanisms responsible for CD8-to-CD4 lineage conversion naturally at a clonal level will greatly benefit therapeutic translation of MHCI-restricted CD4 T cells, particularly MHCI-T_reg_ cells.
